# Mice Selected for Acute Inflammation Present Altered Immune Response during Pristane-Induced Arthritis Progression

**DOI:** 10.1155/2018/1267038

**Published:** 2018-10-08

**Authors:** Mara A. Correa, Andrea Borrego, José R. Jensen, Wafa H. K. Cabrera, Michele Barros, Iana S. S. Katz, Tatiane Canhamero, Monica Spadafora-Ferreira, Jussara G. Fernandes, Nancy Starobinas, Orlando G. Ribeiro, Olga M. Ibañez, Marcelo De Franco

**Affiliations:** ^1^Laboratório de Imunogenética, Instituto Butantan, São Paulo, Brazil; ^2^Laboratório de Imunologia Experimental, Instituto de Ciências Biomédicas (USP), São Paulo, Brazil; ^3^Seção de Diagnóstico, Instituto Pasteur, São Paulo, Brazil

## Abstract

Mouse lines selected for maximal (AIRmax) or minimal acute inflammatory reaction (AIRmin) were used to characterize the immune response and the influence of genetic background during pristane-induced arthritis (PIA). Susceptible AIRmax mice demonstrated exacerbated cellular profiles during PIA, with intense infiltration of lymphocytes, as well as monocytes/macrophages and neutrophils, producing higher levels of IL-1*β*, IFN-*γ*, TNF-*α*, IL-10, total IgG3, and chemokines. Resistant AIRmin mice controlled cell activation more efficiently than the AIRmax during arthritis progression. The weight alterations of the spleen and thymus in the course of PIA were observed. Our data suggest that selected AIRmax cellular and genetic immune mechanisms contribute to cartilage damage and arthritis severity, evidencing many targets for therapeutic actions.

## 1. Introduction

Heterogeneous mice selected for maximal (AIRmax) and minimal (AIRmin) acute inflammatory reaction are useful models for studying cellular and genetic mechanisms involved in arthritis susceptibility [1, 2] that may also contribute to the development of rheumatoid arthritis (RA) in humans.

The stages of RA are difficult to be studied in humans. Therefore, several experimental arthritis models have been developed, becoming valuable tools for comprehensive investigation of the pathogenic pathways involved [3–6] and target discovery for preventive or therapeutic strategies [7]. These models include arthritis induced by the injection of zymosan (ZIA), adjuvants (AIA), proteoglycan (PGIA), type II collagen (CIA), or pristane (PIA) [7, 8]. Adjuvants and mineral oils have been described to induce autoimmune/inflammatory syndrome in humans, resulting from a complex interplay between genetic predisposition and environment factors, as observed in many collateral effect postvaccination phenomena and other disorders [9, 10]. 

PIA has proven to be an important experimental model for RA. The natural saturated terpenoid alkane pristane (2,4,6,10-tetramethylpentadecane) induces an acute inflammation followed by a chronic relapsing phase when introduced into the peritoneal cavity. The reaction is T-cell dependent with edema and articular infiltration of mononuclear and polymorphonuclear cells, anti-dsDNA (double strand DNA), and anti-hsp (heat shock protein) antibodies in serum [11–14].

AIRmax and AIRmin mice were phenotype selected for acute inflammation starting from a polymorphic foundation population (F0), which was established by crossing A, DBA2, P, SWR, CBA, SJL, BALB/c, and C57BL/6 inbred mouse strains. Localized 24-hour leukocyte influx and exudated proteins after subcutaneous injection of polyacrylamide beads were the selection phenotypes used as phenotype indicators 24 h after the subcutaneous injection of polyacrylamide beads [15]. AIRmax and AIRmin lines showed 20-fold and 2.5-fold differences in leukocyte infiltration and exudate protein concentrations, respectively, after selective breeding. In our linkage studies using AIRmax x AIRmin intercrosses, we mapped quantitative trait loci (QTL) for PIA susceptibility on chromosomes 5 and 8 that overlap QTL for experimental arthritis and other autoimmune diseases [2]. Gene expression profiling of paws from arthritic and nonarthritic AIRmax and AIRmin mice showed that among several differentially regulated genes, IL-1beta and CXC chemokines had higher expression in PIA-susceptible AIRmax mice, as well as in macrophages [16].

The selected genetic background in AIRmax modulates the immune responses, with potent stimulation of pro- and anti-inflammatory cytokines, immunoglobulins, and chemokines, which contribute to cartilage damage and consequent arthritis severity. Here we characterized the pristane-induced effects on the activation of immune responses during experimental arthritis in the context of high and low acute inflammation selected phenotypes.

## 2. Methods

### 2.1. Mice

AIRmax and AIRmin mice were bred in the Immunogenetics Laboratory of the Butantan Institute under conventional conditions. All experiments were carried out with equal numbers of male and female mice (2 to 3 months old). All procedures involving animals were approved by the committee for ethics in animal experimentation of Butantan Institute (CEUAIB protocol no. 5343010416).

### 2.2. Pristane-Induced Arthritis

Mice were injected intraperitoneally with two doses of 0.5 mL mineral oil pristane (Sigma Chemical Co., San Diego, CA, USA) at 60-day intervals. Arthritis development was evaluated twice weekly by two independent observers for 180 days by recording maximal clinical scores for each paw and their incidences. Clinical scores, evaluated for each paw, were as follows: 0, no signs of arthritis; 1, mild swelling of the toes or ankle joints; 2, moderate swelling; 3, severe swelling. The maximum score was 12 [17, 18]. The clinical score of each animal was determined by adding the scores of the four limbs (paws) together. Body and organs weight variations were measured after 180 days of pristane injection.

### 2.3. Peritoneal Cells

**Figure 1:**
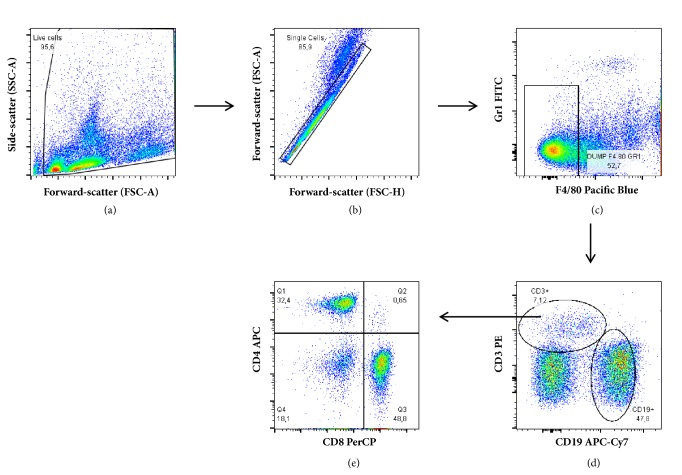
*Flow cytometry gating strategy for lymphoid cells analysis*. Live cells were gated on total cells plot identified by forward scatter (FSC) and side scatter (SSC) properties (a). Cell doublets were excluded by the analysis of the FSC height values over the FSC area values (b). Negative cells to F4-80 and Gr1 markers were selected (c) and then T and B lymphocytes (CD3 and CD19 positive cells, respectively) were analysed (d). In the next step, CD4 and CD8 expression was evaluated inside CD3 positive T cells gate (e).

Peritoneal exudate cells were obtained from control or experimental mice by washing their peritoneal cavities with 5 mL of ice-cold serum-free RPMI1640 medium. The cells from individual mice were centrifuged (160 x g, 10 min, 4°C), washed in cold PBS, and subsequently adjusted to 1x10^7^ cells/mL. Viable cells were counted in a haemocytometer chamber by trypan blue exclusion and then labelled for flow cytometry assay (Figure 1).

### 2.4. Flow Cytometry

Peritoneal inflammatory cells (10^6^) were transferred to 12 x 75 mm polypropylene tubes and centrifuged (300 x g, 10 min). The cell pellets were stained with fluorophore-conjugated antibodies specific for the cell surface markers F4/80, Gr-1, CD11b, MHC class II, CD19, CD3, CD4, and CD8 (BD Biosciences) for 30 minutes at 4°C in the dark. After washing with FACS buffer (PBS + 1% FBS), cells were acquired by a FACSCanto II flow cytometer using BD FACSDiva software (BD Biosciences). Unstained samples were prepared for cell size assessment. Data was analysed using FlowJo v.7.5.5 (Tree Star, Ashland, OR, USA).

### 2.5. Cytokine Assay in the Serum

The levels of 8 cytokines (IL-1*β*, IL-6, IL-10, IL-17, IFN-*γ*, TNF-*α*, MIP-2 (CXCL-2), and GM-CSF) were determined in serum at each time point using Milliplex™ MAPkits (Millipore, Billerica, MA, USA). The kits were used according to the manufacturer's instructions. The data were analysed using Bio-Plex Manager software version 4.1 (Bio Rad, Hercules, CA, USA). Standard curves ranged from 3.2 to 10,000 pg/mL. The lower limits of detection for each cytokine were as follows: 15.2 pg/mL for IL-1*β* and GM-CSF; 3.2 pg/mL for IL-6, IL-10, IL-17, IFN-*γ*, and TNF-*α*; 81 pg/mL for MIP-2 (CXCL-2).

### 2.6. Antibody Detection

The levels of 6 immunoglobulins (IgG1, IgG2a, IgG2b, IgG3, IgM, and IgA) were determined in diluted serum (1 : 25000) at day 180 after PIA induction using Milliplex™ MAPkits (Cod. #MGAMMAG-300k) (Millipore, Billerica, MA, USA). The kits were used according to the manufacturer's instructions. The data were analysed using Bio-Plex Manager software version 4.1 (Bio Rad, Hercules, CA, USA). The lower limits of detection for each immunoglobulin were as follows: 7.89 ng/mL for IgA; 3.25 ng/mL for IgG1; 2.74 ng/mL for IgG2a; 3.16 ng/mL for IgG2b; 2 ng/mL for IgG3; and 2.1 ng/mL for IgM.

### 2.7. Statistical Analysis

Data showing normal distributions are presented as means ± s.d. and the statistical significance between the experimental and control mice of both strains was established by ANOVA, followed by Bonferroni's post-test, using GraphPad Prism 5.0 software (GraphPad Software Inc., San Diego, CA, USA). Values of P < 0.05 were considered as statistically significant. Comparisons of survival significant differences were made using the Log-rank (Mantel-Cox) test.

## 3. Results

### 3.1. Survival Curve and Arthritis Incidence

**Figure 2:**
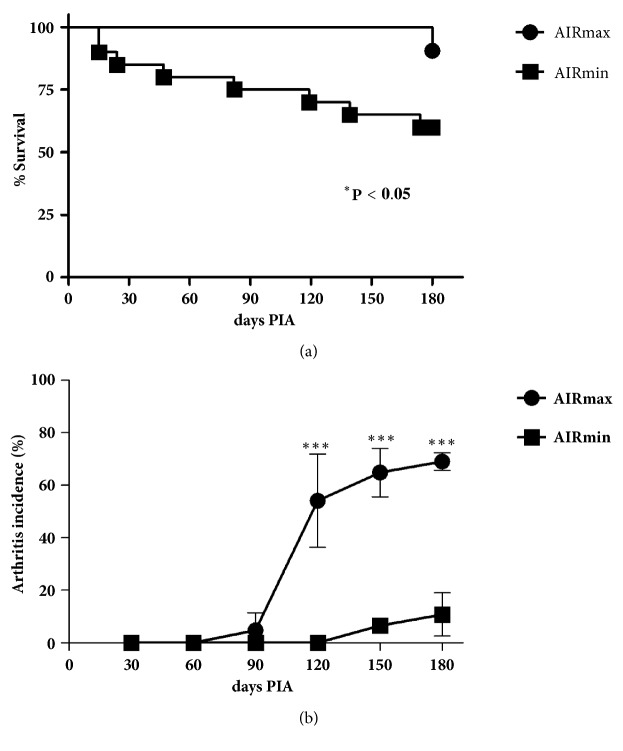
*Survival curves (a) and arthritis incidence in AIRmax and AIRmin mice (b)*. AIRmax and AIRmin mice were injected two i.p 0.5 mL pristane injections and were observed for 180 days. Survival curves: AIRmax (N= 21) and AIRmin (N= 20). The data are presented as mean ± SD. Log-rank (Mantel-Cox) Test (^∗^p < 0.05), in comparison to AIRmin group. Arthritis incidence: AIRmax (N= 33) and AIRmin (N= 32). The data are presented as mean ± SEM and three different experiments (*∗*) p < 0.05; (*∗∗*) p < 0.01; (∗∗∗) p < 0.001.

In the 180-day period of PIA induction, AIRmin mice showed lower survival (60%) than AIRmax (90%, Figure 2(a)), while none of the noninjected controls died. The kinetics of PIA development was very different. AIRmax group already presented clinical disease signs (edema and/or erythema) 120 days after pristane injection. AIRmax mice were significantly (p<0.001) more susceptible (180 d - 69%) than AIRmin mice (180 d – 10%) (Figure 2(b)) evidencing the influence of inflammatory background in PIA. Both lines did not spontaneously develop arthritis and there is no difference between male and female mice.

### 3.2. Effect of Pristane in Body and Organs Weight

**Figure 3:**
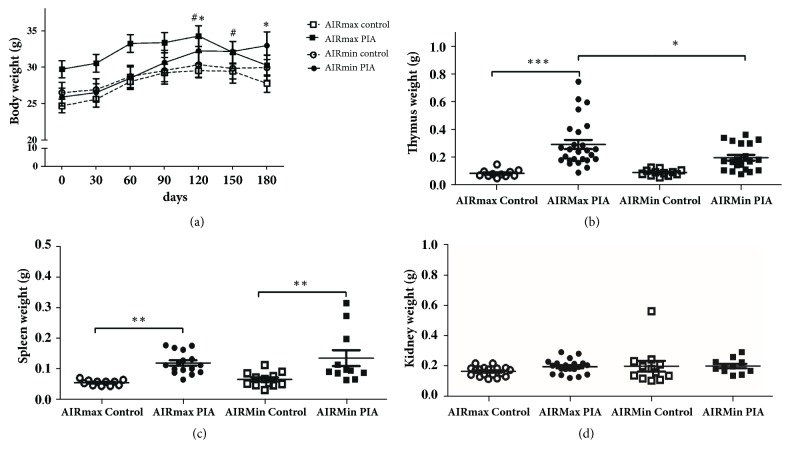
*((a)–(d)) Evolution of body and specific organs weight during pristane-induced arthritis.* Changes in weight mass of AIRmax and AIRmin mice were examined at 180 days after pristane injection. The data are presented as mean ± SEM and three different experiments. AIRmax (N= 33) and AIRmin (N= 32). (*∗*) p < 0.05 AIRmin PIA between 0 d vs. 120 d and 180 d; (#) p < 0.05 AIRmax control between 0 d vs. 120 d and 150 d.

Body weight variation after 180 days of pristane injection is illustrated in Figure 3(a). Significant weight alterations starting at 120 days were observed among the groups, with AIRmax mice submitted to pristane protocol showing highest weight decrease. Spleen, kidney, and thymus weight were also checked (Figures 3(b)–3(d)). As can be observed, AIRmax thymus was heavier than AIRmin thymus after pristane treatment. Similar profile was observed for spleen weight. However, kidney weight did not show alterations in both lines after PIA.

### 3.3. Serum Cytokines after PIA

**Figure 4:**
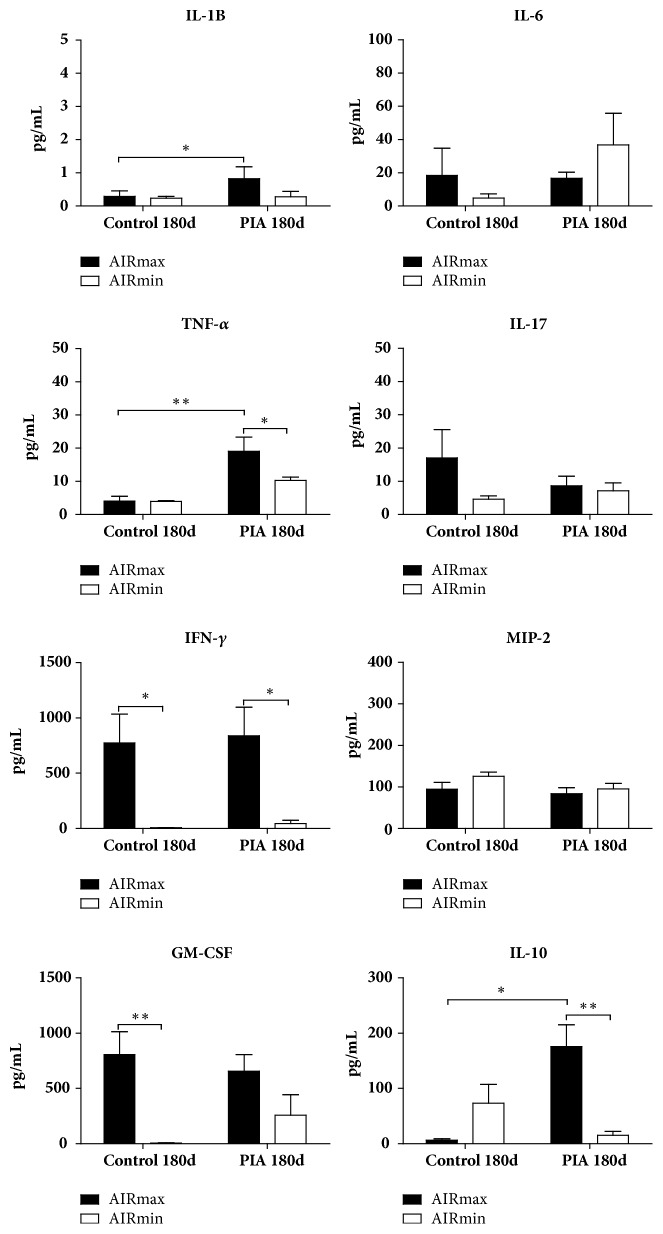
*Time course of changes in cytokine concentrations in pristane-treated mice*. Serum cytokines levels were assessed during arthritis development (0 and 180 days). Reactions were carried out in six biological replicates and the results are expressed as the mean ± SEM. AIRmax (N= 6) and AIRmin (N= 6). (*∗*) p < 0.05; (*∗∗*) p < 0.01.

Serum levels of TNF-*α*, IL-1*β*, and IL-10 were significantly higher in AIRmax than AIRmin mice after 180 days, and higher levels of IFN-*γ* were observed in this same group and period; IFN-*γ* and GM-CSF increased on day 180 in the AIRmax control group (Figure 4).

### 3.4. Autoantibodies after PIA

**Figure 5:**
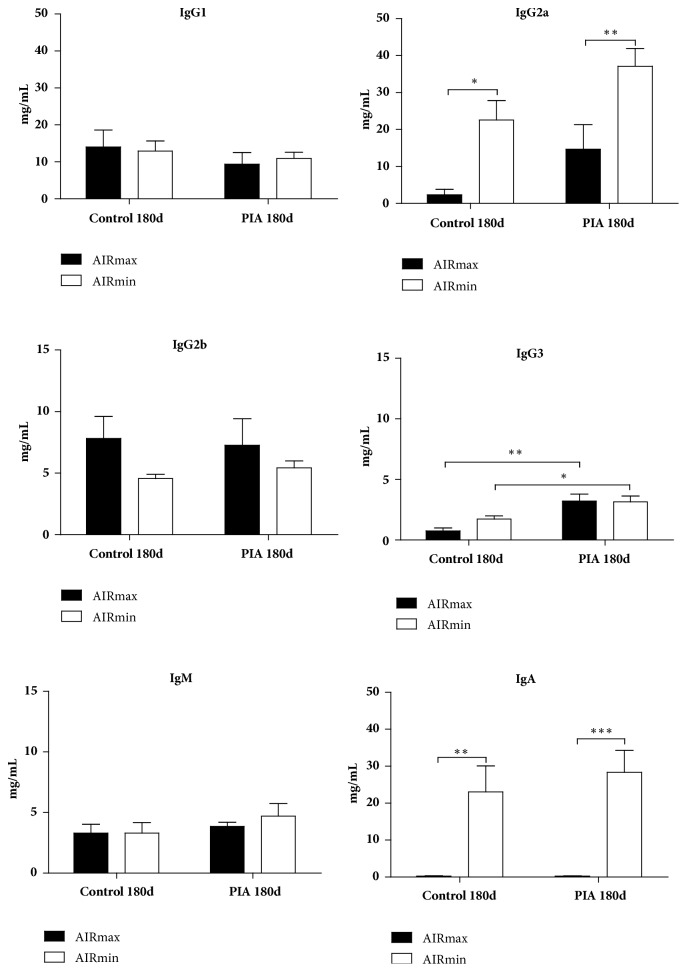
*Time course of changes in immunoglobulin concentrations in pristane-treated mice*. Immunoglobulin levels in serum were assessed during arthritis development (0-180 days). Reactions were carried out in six biological replicates and the results are expressed as the mean ± SEM. AIRmax (N= 6) and AIRmin (N= 6). (*∗*) p < 0.05; (*∗∗*) p < 0.01; (∗∗∗) p<0.001.

Immunoglobulin productions (IgG1, IgG2a, IgG2b, IgG3, IgM, and IgA) were determined in the serum at 180 days after pristane injection, using Multiplex assays, using the same time line protocol. IgG2a and IgA levels significantly increased at 180 days in AIRmin mice (control or PIA group); IgG3 increased only at 180 days PIA in AIRmax and AIRmin mice (Figure 5).

### 3.5. Flow Cytometry Analysis

**Figure 6:**
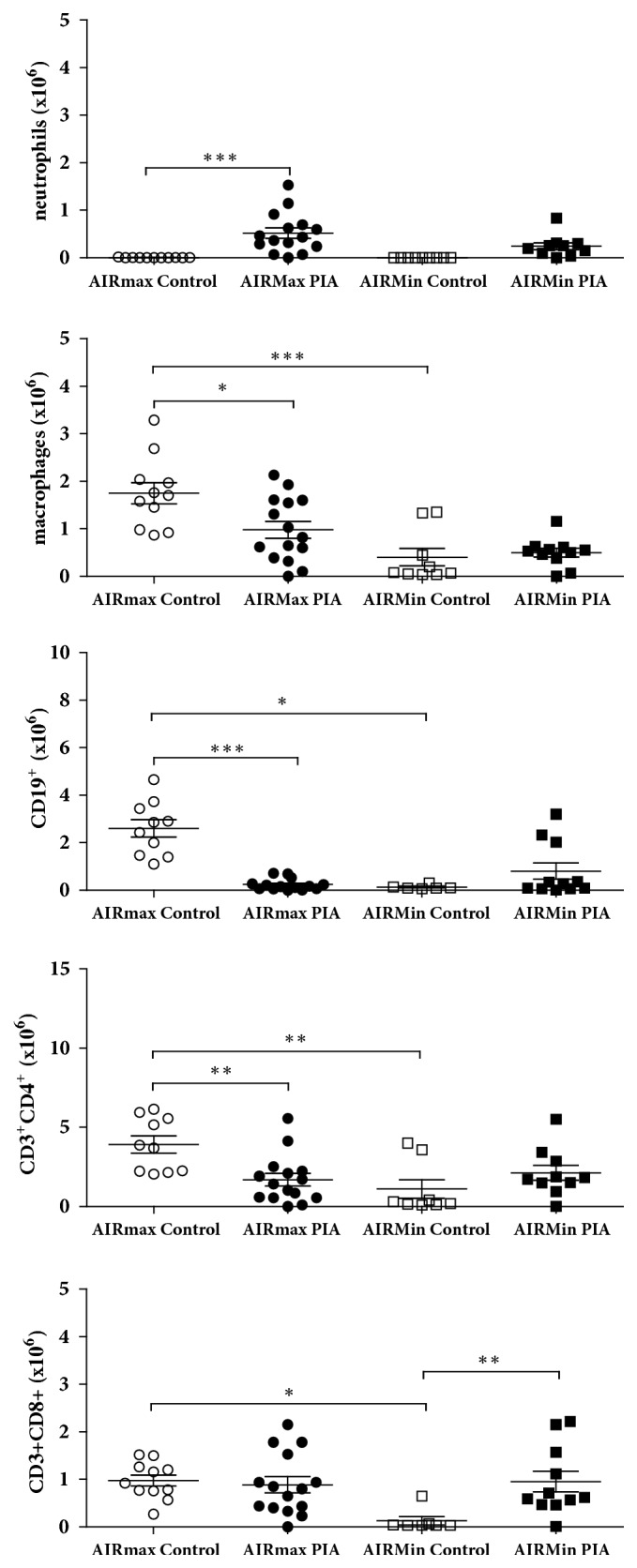
*Flow cytometry analysis of peritoneum cavity of AIRmax and AIRmin during PIA.* Analysis of peritoneum cells from a representative arthritis and control mice at 180 days after pristane injection is shown. The results are expressed as the mean ± SEM. AIRmax (N= 27) and AIRmin (N= 20). (*∗*) p < 0.05; (*∗∗*) p < 0.01; (∗∗∗) p < 0.001.

**Figure 7:**
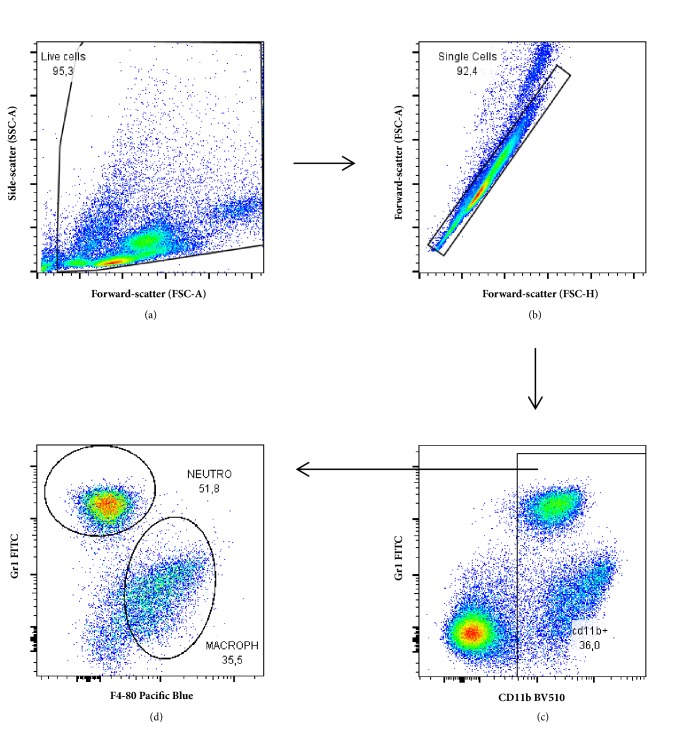
*Flow cytometry gating strategy for myeloid cells analysis.* Live cells were gated on total cells plot identified by forward scatter (FSC) and side scatter (SSC) properties (a). Cell doublets were excluded by the analysis of the FSC height values over the FSC area values (b). Positive cells to CD11b marker were selected (c) and then macrophages and neutrophils (F4-80 and Gr1 positive cells, respectively) were gated on CD11b positive selection (d).

To evaluate the local effect of pristane, the numbers of neutrophils, macrophages, and lymphocytes (TCD4^+^, TCD8^+^, and B cells CD19^+^) were determined in peritoneum cavity at day 180. The neutrophil numbers were higher in AIRmax PIA than control group. However, a high number of macrophages, B cells, TCD4, and TCD8 cells were present in the AIRmax control but not in the AIRmin control and AIRmax PIA. Moreover, TCD8+ cells increased after pristane stimulus in AIRmin mice during PIA (Figures 6 and 7).

## 4. Discussion

Failure to tolerate self-antigens triggers autoimmunity. Major environmental factors are associated with the early stage of development of autoimmunity, for example, infectious agents, vaccines, drugs, tobacco, and stress [19–22]. Genetic interaction and environmental and hormonal factors during the pathogenesis may overlap the aetiology of autoimmunity, making it difficult to understand the mechanisms that lead to the breakdown of immune system tolerance [22, 23]. Mineral oil and adjuvants are involved in many autoimmune diseases resulting from a complex link among high inflammatory conditions, T and B cells altered developments and genetic predispositions [9, 10]. We investigated here whether the differences in the susceptibility to experimental oil induced arthritis of AIRmax or AIRmin mice could be attributed to variations on genetic background of these two mouse lines.

Several models have been developed to reproduce the effects of arthritis in humans. Among these models is the pristane-induced arthritis (PIA) protocol. This is characterized as a chronic inflammatory disease, with late onset and progression ranging from 60 to 200 days according to the experimental murine model used [12]. The main histological features are synovial hyperplasia, cartilage erosion, and pannus formation [24, 25]. These characteristics are observed in BALB/cJ and DBA/1 strains after pristane injection [14]. Previous reports demonstrated that the presence of* Slc11a1 S* allele increased the incidence and severity of PIA in AIRmaxSS, suggesting that this gene could interact with inflammatory loci to modulate PIA [18]. We investigated the effects of* Slc11a1* alleles on the activation of phagocytes during PIA. Our results with AIRmaxSS mice showed differential peritoneal macrophage gene expression profiles during PIA, with higher expression and production of H_2_O_2_, NO, IL-1b, IL-6, TNF-a, and several chemokines. The presence of the* Slc11a1* R allele, on the other hand, diminished the intensity of macrophage activation, restricting arthritis development [16]. Pristane, hexadecane, squalene, and mineral oil also induce arthritis in Lewis and Dark Agouti rats. However, pristane, besides being described as an inducer of arthritis, may function as an inducer of lupus depending on the lineage of mice and rats [26–28]. In BALB/c and SJL mice the deposition of mesangial immunocomplexes is followed by subendothelial lesions consistent with diffuse nephritis-proliferative lupus. In general, lupus arthritis is not erosive. These individuals present an overlap, that is, overlapping syndromes with characteristics of arthritis and lupus. Pristane may further induce immunocomplex-mediated glomerulonephritis in BALB/c and SJL, which develop glomerular IgG and complement depot, cell proliferation, and proteinuria; C57BL/6 develop the disease with low severity. Treatment with pristane significantly increased levels of IgG2a and IgA immunoglobulins in AIRmin animals after 180 days of PIA (Figure 5). Vigar and collaborators [1] also observed significant amounts of IgG2a in the serum of AIRmin animals over the 120-day PIA kinetics. Glomerulonephritis could play a role in the survival rates observed in these mice (Figure 2(a)) and the immunocomplexes deposition in the kidney of these animals is now under investigation. Other studies show that the frequency of selective IgA deficiency in patients with chronic arthritis is between 2% and 4% and the percentage of the prevalence of this immunodeficiency in patients with juvenile systemic lupus erythematosus is from 1% to 4% [29, 30]. On the other hand, PIA AIRmax animals presented a significant increase of IgG3 in the serum at 180 d, compared to their control group. We did not detect differences in IgG1, IgG2b, and IgM levels (Figure 5).

Pristane oil injection significantly increases TNF*α*, IFN-gamma, and IL10 serum cytokines in AIRmax mice after 180 days, which are higher than that observed in the AIRmin lineage. The AIRmax control group also secreted high levels of IFN-gamma and GM-CSF (Figure 4), suggesting that high acute inflammation genetic background could prompt the spontaneous response of both cytokines. Cells collected from the AIRmax and AIRmin mice peritoneum at day 180 after PIA induction were labelled with specific antibodies against surface molecules for granulocytes (GR1^+^ CD11b^+^), T lymphocytes (CD3^+^ CD4^+^ and CD3^+^ CD8^+^), B lymphocytes (CD19^+^), and macrophages (F4/80^+^ CD11b^+^ CD11c^−^). The mononuclear and granulocyte infiltrates induced by pristane in the peritoneal cavity in the AIRmax susceptible strain were higher than the AIRmin line, with a higher frequency of macrophages, T lymphocytes, and neutrophils 180 days after pristane treatment. A significant increase in CD8^+^ T cells in AIRmin was observed at 180 days. Persistent inflammation in AIRmin peritoneum could promote bacterial translocation, inducing the CD8^+^ T cells increase together with the significant amounts of IgG2a observed in the serum of these animals, which might favour the higher mortality in this line.

AIRmax mice started to develop arthritis after 90 days after PIA, while AIRmin mice are completely resistant at that time (Figure 2(b)). It should be noted that the two lines analysed do not develop arthritis spontaneously. These data reproduced those previously published by our group [1]. Macroscopic analysis showed a significant increase of the thymus and spleen masses from AIRmax and AIRmin mice after 180 days of pristane injection. However, the AIRmax thymus is significantly heavier than the AIRmin thymus, characterized as follicular thymic hyperplasia, suggesting that this difference may also favour the susceptibility to arthritis due to dysregulation of the central tolerance performed by the thymus. Our hypothesis is that thymic selection in AIRmax background under exacerbated inflammatory conditions is altered, affecting the development/proliferation of T lymphocytes, which contributes to the arthritis susceptible phenotype. These changes are also observed in most patients with myasthenia gravis, and in some cases they occur in other autoimmune diseases such as systemic lupus erythematosus and rheumatoid arthritis [30].

In summary, AIRmax mice showed differential susceptibility to PIA with neutrophils, macrophages and lymphocytes altered profiles in peritoneal cavity, presenting higher expression and production of IL-1*β*, IFN-*γ*, TNF-*α*, IL-10, and IgG3. The AIRmin genetic background, on the other hand, favours the low intensity of cellular activations, restricting arthritis development. This study suggested that the AIRmax and AIRmin PIA model results, when compared to those obtained in inbred mice, evidence new possible therapeutic targets and the interference of genetic background in the mechanisms underlying arthritis susceptibility and severity.

## Data Availability

The data files used to support the findings of this study are available from the corresponding author upon request.
